# Concomitant Congenital Diaphagmatic Hernia (CDH) and bilateral bacterial glomerulonephritis in a pet chinchilla (*Chinchilla lanigera*)

**DOI:** 10.1186/s12917-021-03085-4

**Published:** 2021-12-03

**Authors:** Alessandro Vetere, Mara Bertocchi, Emanuele Moggia, Igor Pelizzone, Francesco Di Ianni

**Affiliations:** 1 Clinica Veterinaria Modena Sud, Piazza dei Tintori 1, 41057 Spilamberto, MO Italy; 2 Department of Veterinary Science, University of Parma, Strada del Taglio 10, 43126 Parma, PR Italy; 3 Ambulatorio Veterinario Levante, Via Alberto Salietti, 6, 16043 Chiavari, GE Italy; 4 Ambulatorio Veterinario Belvedere, Via Pietro Bembo 12, 42123 Reggio Emilia, RE Italy

**Keywords:** Diaphragmatic hernia, Glomerulonephritis, Chinchilla

## Abstract

**Background:**

The domestic chinchilla has been descended from *Chinchilla lanigera* (long-tailed Chinchilla) or *Chinchilla chinchilla* (short-tailed Chinchilla). Both species of chinchilla are currently listed as endangered by the IUCN Red List of Threatened Species. Over the past 20 years, they have spread as pets and overall knowledge about their care is improving. The present case report describes a congenital diaphragmatic hernia in a *Chinchilla lanigera*.

**Case presentation:**

A 1-year-old, 420 g female chinchilla (*Chinchilla lanigera*) was presented for clinical examination due to 2 days haematuria episodes and anorexia. A complete haematological analysis was performed, showing a moderate neutrophilia and severe renal involvement. X-rays showed severe intestinal meteorism affecting mostly the cecum, and a soft tissue density mass with translucent areas located in the caudal thorax, making it hard to distinguish the cardiac silhouette. A barium swallow (barium sulfate) was performed and after 20 min, radiograms were performed again, showing part of the stomach dislocated in thorax. Ultrasound was also carried out, confirming the partial stomach herniation into the thoracic cavity and a severe nephropathy. The patient was euthanized according to the owner’s wish and a complete necropsy was performed. The diagnosis was congenital diaphragmatic hernia concomitant to a severe bilateral bacterial glomerulonephritis.

**Discussion and conclusions:**

Diaphragmatic hernias can be either congenital or acquired. About CDHs in pet chinchillas, literature is still lacking. In this patient there was no history of previous traumas. No scar tissue or thickening involved margins of the pathological diaphragm window at the necropsy, supporting the hypothesis of a congenital defect. Glomerulonephritis most often results from immune-mediated mechanisms, generally after the deposition of soluble immune complexes within the glomeruli. This mechanism is favoured by a prolonged antigenemia that could occur during specific viral infections, chronic bacterial infections, chronic parasitism, autoimmune diseases and neoplasia. Few cases of nephritis are described in chinchillas (*Chinchilla lanigera*), mostly related to bacterial sepsis or less commonly involving fungi. The evidence of bacterial aggregates in kidneys at the histopathology, confirmed the infective aetiology. No relationship between the diaphragmatic hernia and glomerulonephritis was found in this report.

## Background

Congenital diaphragmatic hernia (CDH) is a congenital abnormality consisting of a defect in the diaphragm developed during the embryogenesis [1]. In mammals, the diaphragm muscle is essential for the respiratory function because it contracts and thereby induces expansion of the thorax, inducing a negative pressure to help draw air into the lungs [1]. In contrast, on exhalation the diaphragm helps expelling the air from the lungs by its relaxation [1]. The diaphragm physically separates the thorax from the abdominal cavity. This characteristic is altered in CDH. CDHs in small animals are usually considered to be chance embryonic anomalies rather than heritable defects, while traumatic diaphragmatic hernias occur due to blunt abdominal traumas, like car accidents, falls or other traumatic abdominal injuries [1–3]. Glomerulonephritis constitutes an important category of renal diseases in animals most often resulting from immune-mediated mechanisms [1], generally after the deposition of soluble immune complexes within the glomeruli, or caused by the formation of antibodies directed against antigens within the glomerular basement membrane [4–7]. The present paper describes a case of concomitant CDH and a bilateral bacterial glomerulonephritis in a pet chinchilla (*Chinchilla lanigera*).

## Case presentation

**Fig. 1:**
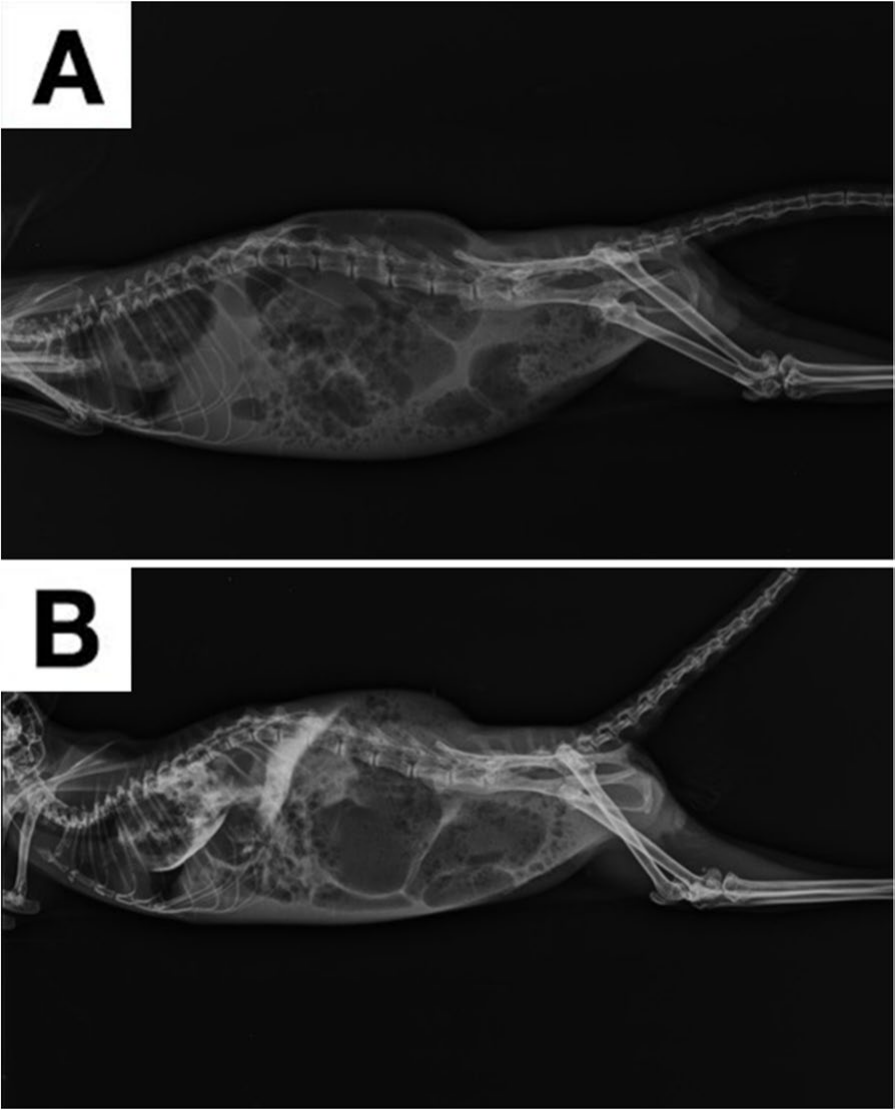
Lateral projection of the patient before (**A**) and 20 min after (**B**) the baritate meal. The evidence of half of the stomach dislocated into the thoracic cavity made possible the diaphragmatic hernia diagnosis

**Fig. 2:**
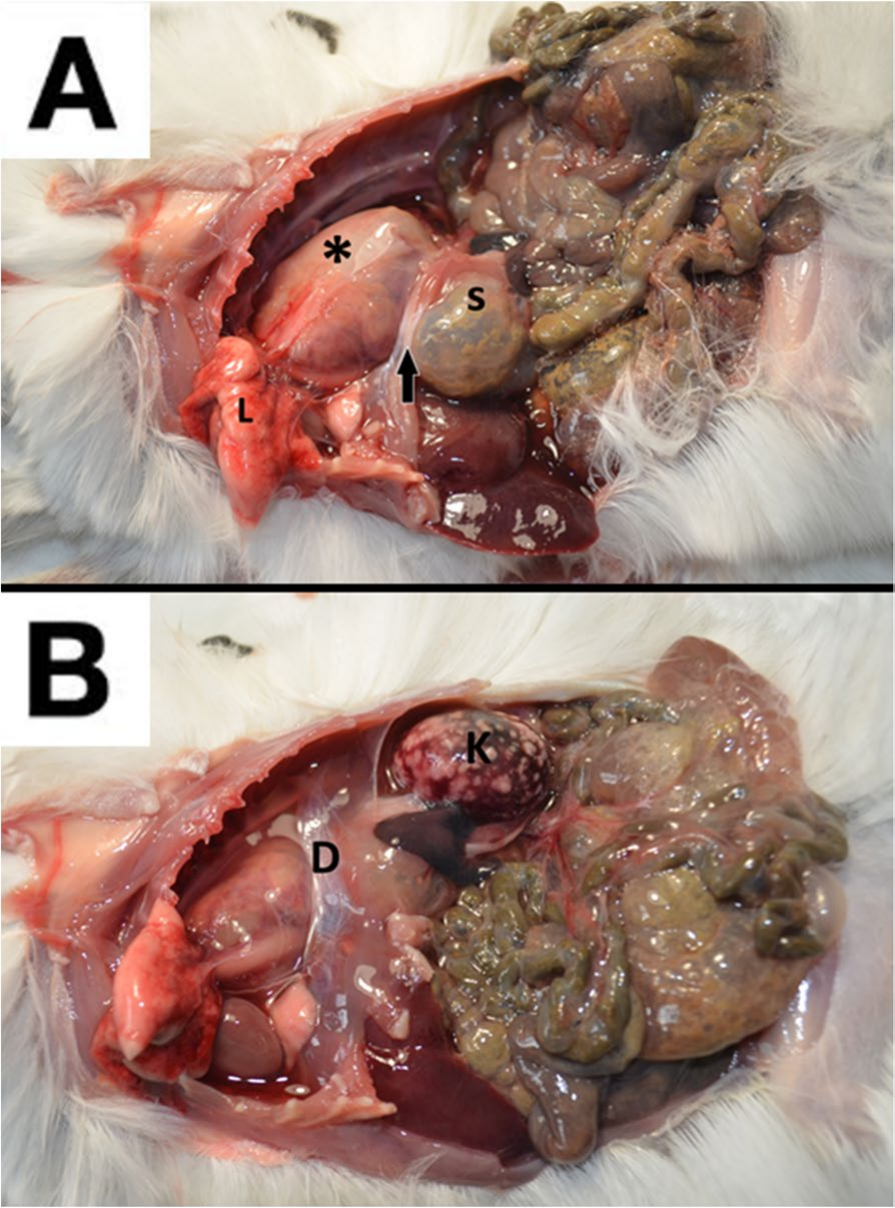
Necropsy. **A**. The stomach (S) protruding into the chest cavity. The herniated portion (asterisk) represents almost the 60% of the entire organ. Black arrow: diaphragm muscle. L: lung. **B** The kidney (K) is involved by yellowish disseminated lesions, protruding the organ surface. D: Diaphragm, central tendon portion

**Fig. 3:**
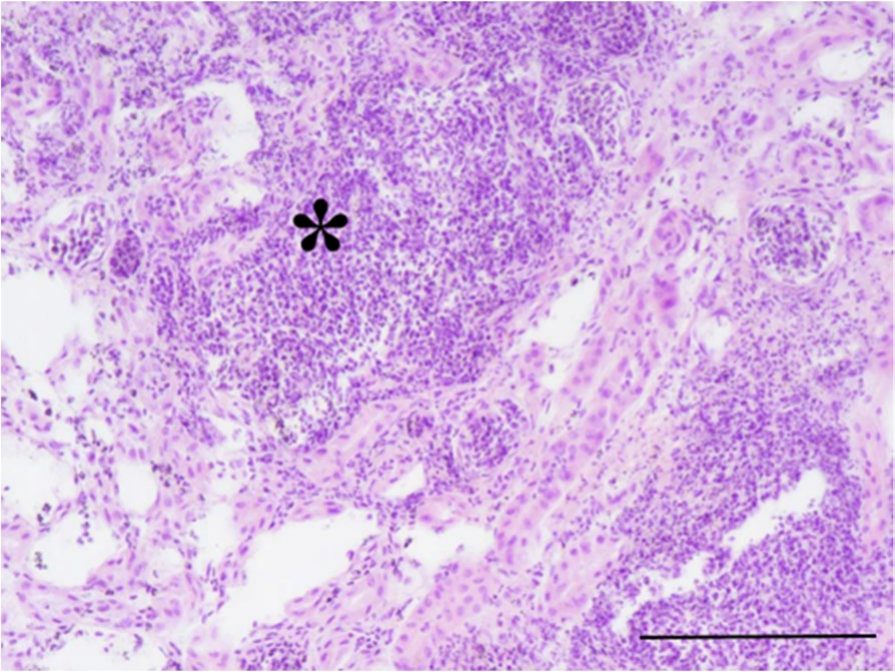
Histological section of the kidney involved in the pathological process. A severe inflammatory response is evident in the histological section of the renal parenchyma. Abundant degenerated and non-degenerated heterophil aggregates are evident (asterisk). Normal *architecture* is almost totally *replaced* by a suppurative process. Hematoxilin-Eosin Stain, 200x. Scale bar 200 μm

A 1-year-old, 420 g female chinchilla (*Chinchilla lanigera*) was presented for clinical examination due to 2 days haematuria episodes and anorexia. Husbandry and diet were adequate for the species and no recent traumas were reported by the owner. Physical exam revealed slight tachypnea compared to the reference values [90 breaths/min (bpm)] [8] and increased rectal temperature (40,2 °C). A biochemical analysis was performed, showing severe renal involvement. (Table 1) [8]. The complete blood count (CBC) parameters showed also a moderate heterophilia compared to the species reference values (Table 2) [8]. The CBC count was performed using Idexx ProCyte® Dx analyzer [IDEXX Laboratories Italia, S.r.l. Via Canova 25, 20,147 Milano, Italia]. Radiograms were also performed, showing severe intestinal meteorism affecting mostly the cecum and a soft tissue density mass with translucent areas (air) located in the caudal thorax, making it hard to distinguish the cardiac silhouette (Fig. 1A) Suspecting a diaphragmatic hernia, a barium swallow (barium sulfate) was performed at the dosage 2% of the body weight (60% weight/volume micro pulverized barium sulphate and water) [9] gently administered with a 2,5 ml syringe, orally. After 20 min, other lateral and ventro-dorsal radiograms were performed, showing the half of the stomach dislocated into thorax (Fig. 1B). Abdominal ultrasound was also performed confirming the partial stomach herniation in the thoracic cavity and also a severe nephropathy involving both kidneys. Bladder was empty at the ultrasonography, so the urinalysis couldn’t be performed. The patient was euthanized after the owner’s consent. Gross pathological investigation, performed by a certified pathologist, showed half of the stomach displaced into thorax without any signs of recent traumas (Fig. 2A). A slight presence of adherences between pleural surface and stomach serosa were visible (Fig. 2A). No scar tissue or thickening were highlighted at the margins of the pathological diaphragm window, which was set between muscular pillars in the central tendon supporting the hypothesis of a congenital defect. Both kidneys were affected by disseminated yellowish miliary lesions, deforming the kidney’s surface (Fig. 2B). A small fragment of kidney parenchyma was sampled for bacterial culture; unfortunately, bacterial isolation was not possible due to the large amount of different bacteria grown on the blood agar plate (BAP). Both kidneys were sampled and 10% buffered formalin fixed for the histology. Olympus CX-43 microscope [Olympus Italia SRL, Via Modigliani 45, Segrate, 20,090, MI, Italia] equipped with Olympus EP-50 camera [EPview software, 5 Megapixel, 2592 × 1944 Resolution photograph (Pixel)] was used to capture histological images. The histopathologic diagnosis was acute, severe bilateral bacterial glomerulonephritis, with 90% of parenchyma involved and infiltrated by degenerated heterophils. Bacterial aggregates and necrosis were also present in both kidneys. The diagnosis was CDH concomitant to a severe bilateral bacterial glomerulonephritis (Fig. 3).

**Table 1:** Comparison between biochemical values of the patient and the reference values. Abnormal findings are bold

Analytes	Patient	Reference Values^a^
AST (U/l)	88	96
Total protein (g/dL)	3.9	3.8–5.6
Albumin (g/dL)	2.2	2.3–4.1
Globulin (g/dL)	1.7	0.9–2.2
Creatine kinase (U/l)	0.7	0.6–1.0
LDH (U/l)	312	–
Phosphorous (mmol/l)	**3.5**	1.3–2.6
Calcium (mmol/l)	1.4	1.4–3
Potassium (mmol/l)	3.5	3.3–5.7
BUN (mg/dL)	**58**	17–45
Creatinine (mg/dL)	**5.2**	0.4–1.3
Glucose (mg/dL)	120	109–193
Triglycerides (mg/dL)	43	–

^a^ [8]

**Table 2:** Comparison between the CBC parameters of the patient and the reference values. Abnormal findings are bold

Parameters	Patient	Reference values^a^
WBC × 10^3^/μl	**16.1**	5.4–15.6
RBC × 10^6^/μl	5.7	5.6–8.4
Hb (g/dl)	12.1	11.8–14.6
MCV (fl)	54.4	–
MCH (pg)	21.2	–
MCHC (%)	39	–
HCT (%)	31	27–54 (PCV)
Neutrophils (%)	**62**	39–54
Eosinophils (%)	1	0–5
Basophils (%)	0	0–1
Monocytes (%)	3	0–5
Lymphocytes (%)	34	45–60

^a^ [8]

## Discussion and conclusions

Diaphragmatic hernias occur when an entire or a portion of abdominal organs enter the thoracic cavity through a pathological opening involving a defect of the diaphragm muscle [1]. Diaphragmatic hernias are either congenital or acquired [1]. CDHs in small animals are usually considered to be chance embryonic anomalies rather than heritable defects [2, 3] while traumatic diaphragmatic hernias occur due to blunt abdominal traumas [1, 3] like falls, twists and car accidents [3]. The owner reported that the chinchilla had “deep breath” since his birth. He thought that this behaviour was normal, since he ate, did sand baths and interacted with her without any problem. For this reason, he hasn’t been seen by a veterinarian. Glomerulonephritis most often results from immune-mediated mechanisms, generally after the deposition of soluble immune complexes within the glomeruli. This mechanism is favoured by a prolonged antigenemia that enhances the formation of soluble immune complexes, that could occur during specific viral infections, chronic bacterial infections, chronic parasitism, autoimmune diseases, and neoplasia. Less commonly glomerulonephritis is caused by the formation of antibodies directed against antigens within the glomerular basement membrane, implying an autoimmune condition. Antibodies to the basement membrane bind and damage the glomerulus through fixation of complement and resulting leukocyte infiltration [10]. Glomerulonephritis is responsible for substantial morbidity and mortality in many species of animals [4]. Few cases of nephritis are described in Chinchillas (*Chinchilla lanigera*) [5–7]. Five cases of nephritis by *Salmonella* spp. and *Leptospira interrogans serovars ictero-haemorrhagiae* and *pomona* were reported by Martino et al. [5]. A suppurative tubulonephritis was described as a result of septicaemia and subsequent disseminated intravascular coagulation caused by attaching-effacing *Escherichia coli* (AEEC) [6]. Only one case of glomerulonephritis is reported due to *Histoplasma capsulatum*. This fungus exists in a hyphal form in environments with high moisture and organic content. It is transmitted by inhalation of airborne spores and then it invades tissues via a single budding yeast phase. In this female Chinchilla, the kidney presented multiple focal areas of glomerular nephritis characterised by an accumulation of polymorphonuclear cells in the proximal tubules. Aggregates of *H. capsulatum* yeast cells were present in glomerular epithelioid cells [7]. In this report, no scar tissue or thickening involved margins of the pathological diaphragm window, set between muscular pillars in the central tendon supporting the hypothesis of a congenital defect. The severe increase of creatinine, the slight increase of phosphorus and the moderate increase of blood urea nitrogen (BUN) were related to the severe renal damage [11]. The moderate heterophilia is probably related to the urinary bacterial infection; although the urinalysis wasn’t performed due to the empty bladder, the evidence of bacterial aggregates in kidneys at the histopathology, supported the infective aetiology. The multitude of different bacteria found at the bacteriological analysis has made impossible the identification of the aetiological agent of glomerulonephritis. This finding was attributed to a sample contamination. No relationship between the diaphragmatic hernia and glomerulonephritis was found in this case study.

## Data Availability

All data generated or analyzed during this study are included in this published article [and its supplementary information files].

## Abbreviations

AEEC: Attaching-effacing *Escherichia coli*
AST: Aspartate aminotransferase
bpm: Beats per minute
BUN: Blood urea nitrogen
CBC: Complete blood count
CDH: Congenital diaphragmatic hernia
Hb: Hemoglobin
HCT: Hematocrit
LDH: Lactic Acid Dehydrogenase
MCH: Mean corpuscular hemoglobin
MCHC: Mean corpuscular hemoglobin concentration
MCV: Mean corpuscular volume
min: Minute
RBC: Red blood cells
WBC: White blood cells
